# Notes of the Week

**Published:** 1921-09-17

**Authors:** 


### NOTES OF THE WEEK.

### "OUR DAY" IN LONDON.

Friday, September 30, has been set apart for the celebration of "Our Day" in the city of London, and the following day, Saturday, October 1, for the County of London and adjacent districts. In most parts of the country, too, special efforts are being made to raise money in furtherance of the Red Cross peace programme. The organisation controlled by the Joint Council of the Order of St. John and the British Red Cross Society lends itself admirably to the carrying out of schemes for the improvement of the health of the community, the principle of decentralisation adopted making it possible for every county to base its plans on local needs.

### THE LEICESTER CELEBRATIONS.

As we go to press Leicester is celebrating in a wonderful manner the 150th anniversary of the Royal Infirmary. On Sunday, September 11, a special service of thanksgiving was held in the morning at St. Martin's Cathedral Church, the preacher being the Ven. the Archdeacon of Leicester, and in the afternoon, at the De Montfort Hall, there was a united service of thanksgiving, preceded by an organ recital. On Monday, September 12, at 3.30 p.m., took place a reception by the Chairman of the Board and Mrs. Fielding Johnson at the De Montfort Hall, while on Tuesday and Wednesday the proceeds of a cricket match between representative Leicestershire and Derbyshire elevens were devoted to the Infirmary anniversary fund. For Thursday a pageant has been arranged by Mr. Herbert Pochin and members of the West End Adult School, and on Friday there is the Grand Infirmary Ball at the De Montfort Hall, as arranged by Mrs. Fielding Johnson and Mrs. C. F. Oliver. An interesting evening is expected on Saturday, when at 7.30 a series of five-minute papers, "Then and Now," will be read on various aspects of the town, and medical work in the town and county in 1771 and 1921. The organiser, Mr. Harry Johnson, House Governor and Secretary of the Infirmary, is to be heartily congratulated upon the completeness of the arrangements.

### WELFARE SUPERVISION.

As we go to press a valuable series of lectures and short conferences, organised by the Industrial Welfare Society, is being given at Balliol College, Oxford. On Thursday, September 15, Lord Gorell will deliver the opening address, and on Friday and Saturday the following subjects will be dealt with: "Industrial Psychology," "Economic History," "Camping," "The Uses of Debate," "Educational Possibilities," "English Folk Dances and Folk Music," the respective speakers being Messrs. Eric Farmer, Delisle Burns, Stanford Wood, F. S. Button, Dr. C. W. Kimmins, and Mr. Cecil Sharp. On Sunday the Rev. Robert R. Hyde will give an address on "Welfare at the Cross-Roads" and later in the day and on Monday lectures will be given as follows: "The Attitude of Labour," by Mr. Fred Bramley, "The Employer's Part," by Sir Andrew R. Duncan, and "The Health of the Industrial Worker," by Dr. E. L. Collis. Each lecture will occupy forty-five minutes, and, after a short interval, be followed by one hour's discussion.

### MINERS AND HOSPITAL CONTRIBUTIONS.

Some interesting figures have been published in the *Newcastle Daily Journal* to show the relation of miners' contributions to the cost of the treatment which they receive at the Royal Victoria Infirmary. In a summary statement it is estimated that the contributing workers in Northumberland and Durham receive in treatment the equivalent of about £225 for every £100 which they subscribe, together with the privilege of appointing one governor for every £10 contributed. The cost of the maintenance of in-patients averages £10 per head, and some typical figures are the following:—From the Durham mining community 1,761 in-patients were treated at a cost of £17,610. The contributions of the colliery owners were £1,997, those of the workers £8,947, making a total of £10,944. The deficit to the hospital was therefore £6,665. We understand that the average contribution is less than twopence a week, and that in certain works it is still only one shilling a quarter. If the average could be raised to threepence a week all round, the contributors would still be in the hospital's debt. We should not use this expression in connection with a voluntary hospital did not the workers in the north sometimes speak as if they owned and supported these institutions. If they did, and the amount of their payments were assessed by an actuary, it is estimated that at least sixpence a week would be required. There is no need to be commercial with support given by the other classes of the community; threepence a week all round would do much to meet the present deficit.

### A LOCAL HOSPITAL PAGEANT.

A hospital pageant week is being held as we write at Birkenhead for the benefit of the Borough Hospital, by which an attempt is being made to pay off the debt of £10,000. We hope to have an opportunity of studying the programme of the week's festivities. The result, and the experience gained by Mr. R. H. Gossage, the hospital's secretary, and his assistant, Mr. E. I. Maggs, would be of interest to the committee lately formed in London to consider the scheme for holding a hospital pageant in the Metropolis. If a local effort of this kind can be successfully organised in a town of the size of Birkenhead, it promises well for the larger project of a hospital pageant in London.

### INDIAN GRADUATES' MIDWIFERY.

*Apropos* of the article in The Hospital for September 3 a meeting of the South Indian Medical Union has passed resolutions repudiating "the unjustifiable insinuation" of the General Medical Council, London, that the provisions for midwifery training in India were not efficient, protesting against the Council's proposal to withhold recognition of Indian medical degrees, urging upon the

Government of India the necessity for the formation of an Indian General Council of medical education and registration, and requesting Government to alter the regulations for admission to the I.M.S., so as to render eligible for admission only those who possess either approved Indian medical degrees or diploma, or foreign qualifications recognised as equivalent thereto by the Indian Medical Council to be constituted, and to provide for conducting the I.M.S. examination chiefly in India.

### THE HAMPSHIRE "HOSPITAL COMMITTEES."

The voluntary Hospitals Commission has made a decision in regard to the hospital committee for Hampshire which is interesting because it is likely to be followed in other countries where a similar question occurs. The decision is to form one committee for the county and the county boroughs of Bournemouth and Southampton, and another committee for the county boroughs of Portsmouth because of its size. The decision is probably wise, not only because of the large population of Portsmouth, but also because the hospital finance of the latter is in some respects a special problem since it depends upon the support of the dockyards which, as we have lately pointed out, is for various official reasons not very easy to organise.

### ROSE DAY AND RACE DAYS.

Thanks in part perhaps to the coincidence of the St. Leger meeting with the annual Rose Day held for the benefit of the four Sheffield hospitals, the first results seem to have been very successful. A quarter of a million roses were ordered and bands of nurses from all the Sheffield hospitals were early on the scene. Further supplies, we are informed, had to be procured by telegraph. The Royal Hospital was the headquarters of the sale, and there the chairman of the committee, Lieut.-Col. J. C. Leslie, and Mr. T. H. Williamson, honorary secretary, were at work early and late. In the outlying districts collections were taken last Saturday. It is possible that in one respect the coal strike may have been indirectly helpful. Had it not occurred Rose Day would have been held earlier in the year. Whether the annual collection should in future always be held on the occasion of a race meeting is a point worth consideration by the organising committee. The enthusiasm of hospital staffs on behalf of their institutions has been everywhere a remarkable fact of these difficult times.

### THE ROYAL WATERLOO HOSPITAL.

Attention has been drawn to the suspended building of the new wing of the Royal Waterloo Hospital. The building was begun because the hospital wished to avoid losing some of the old houses which it occupies on the Duchy of Cornwall estate, a loss which would have made a serious inroad into the accommodation. A sum of about £24,000 has been spent on the half-finished building, which will remain incomplete, failing unexpected help, so long as the cost of maintenance is not reduced from its present figure. In October a campaign to raise further money is expected. Perhaps the sight of the forlorn scaffolding is to the public a more impressive sign of financial straits than any statement of unpaid bills. The *Daily Chronicle* has given publicity to the state of affairs, but its very brief statement of the reasons which led to the beginning of the new building require more expansion if they are to be sympathetically appreciated by the general public.

### A SCHOOL DOCTOR ON DAYLIGHT SAVING.

Dr. Clark, the school doctor, Rochdale, in his annual report, criticises the conditions of employment for children in the cotton and woollen mills, and the effect upon their health of the Daylight Saving Act. He says that economic considerations and not the suitability of the employment is the chief concern of the parents, who do not seek advice on the children's fitness for their work, although those with a tendency to phthisis or suffering from respiratory diseases find in the mills unfavourable conditions for their health. He adds that the Daylight Saving Act has not increased the health of school children, who get insufficient rest and sleep because the number of hours of daylight tends to shorten the hours of rest. The effect is seen, he says, in the functional disorders of the heart from which the weak children suffer. The complaint against the parents is as old as the Factory Acts themselves, but it would be interesting to know how far other school doctors can confirm Dr. Clark's opinion on the ill-effects of daylight saving.

### THE CALL OF THE HOSPITAL.

The following, forwarded by a correspondent, himself a witness of the incident, well illustrates to what extent the claim of the voluntary hospital has upon the medical man and how great is the debt of the public—albeit often unrealised—for such self-sacrificing work. A few days ago a doctor in suburban London was attending his surgery patients when he received a telephone message asking his immediate attendance at the local hospital, as a serious accident case had come in; returning to his surgery, the medical man in question told his patients, of whom there was a goodly few, that he could only see any urgent case as he had a call to hospital; this dismissed the majority of those present, together with their fees, and the hon. member of the hospital staff was on his way inside the space of twelve minutes!

### WELCOME BEQUESTS TO GLASGOW HOSPITALS.

Sums amounting to £40,000 have been made to Glasgow hospitals from the residuary estate of the late Miss Mary Hamilton, of Glasgow. Her legacies to charitable institutions, which have already been paid, amount, we understand, to £165,000. The testatrix authorised her trustees to divide the residue of her estate among charities. The distribution will include the following: Glasgow Royal Infirmary, the Western Infirmary, and the Victoria Infirmary, £7,000 each; Glasgow Royal Maternity and Women's Hospital and the Royal Incorporation of Hutcheson's Hospital, £5,000 each; and to the Glasgow Royal Hospital for Sick Children, £2,500. The gifts are doubly welcome not only for their generosity, but because the Glasgow hospitals are in very great need.

